# Supplementary material for: Modelling post-implantation human development to yolk sac blood emergence
Source: Nature. 2023 Dec 13;626(7998):367–76. doi: 10.1038/s41586-023-06914-8 (PMC10849971; doi:10.1038/s41586-023-06914-8)
Supplement: Supplementary file 1 — Supplementary Figs. 1 and 2 and Table 1. Supplementary Fig. 1: Flow cytometry back-gating strategies. Supplementary Fig. 2: Flow cytometry gating strategies. Supplementary Table 1: Oligonucleotides used for RT–qPCR. [file 41586_2023_6914_MOESM1_ESM.pdf]

nature portfolio

https://doi.org/10.1038/s41586-023-06914-8

Supplementary information

Modelling post-implantation human development to yolk sac blood emergence

In the format provided by the authors and unedited

Supplementary Information
2022-01-20991E

Modelling Post-Implantation Human Embryogenesis and Early Hematopoiesis via Engineering a Multilineage Yolk Sac Tissue Niche

Joshua Hislop[1-3], Qi Song[4,5#], Kamyar Keshavarz F.[1-3#], Amir Alavi[4,5#],Rayna Schoenberger[1,3], Ryan LeGraw[2,3], Jeremy Velazquez [2,3], Tahere Mokhtari[1-3], Mohammad Naser Taheri[1-3], Matthew Rytel[2,3], Susana M Chuva de Sousa Lopes[8], Simon Watkins[6,7], Donna Stolz[6,7], Samira Kiani [1-3,9], Berna Sozen[10], Ziv Bar-Joseph[4,5], Mo R. Ebrahimkhani[1-3,9 *]

Affiliations:

1. Department of Bioengineering, Swanson School of Engineering, University of Pittsburgh, Pittsburgh, PA 15261, USA
2. Department of Pathology, Division of Experimental Pathology, School of Medicine, University of Pittsburgh, Pittsburgh, PA 15213, USA
3. Pittsburgh Liver Research Center, University of Pittsburgh, Pittsburgh, PA 15261, USA
4. Computational Biology Department, School of Computer Science, Carnegie Mellon University, Pittsburgh, PA, USA
5. Machine Learning Department, School of Computer Science, Carnegie Mellon University, Pittsburgh, PA, USA.
6. Center for Biologic Imaging, University of Pittsburgh, Pittsburgh, PA, USA.
7. Department of Cell Biology and Molecular Physiology, University of Pittsburgh, Pittsburgh, PA, USA
8. Department of Anatomy and Embryology, Leiden University Medical Center, Einthovenweg, 2333 ZC Leiden, the Netherlands
9. McGowan Institute for Regenerative Medicine, University of Pittsburgh, Pittsburgh, PA 15219, USA
10. Department of Genetics, Yale School of Medicine, Yale University, New Haven, CT, 06510, USA.

*Correspondence: mo.ebr@pitt.edu
[#]These authors contributed equally.

Flow cytometry backgatings and controls

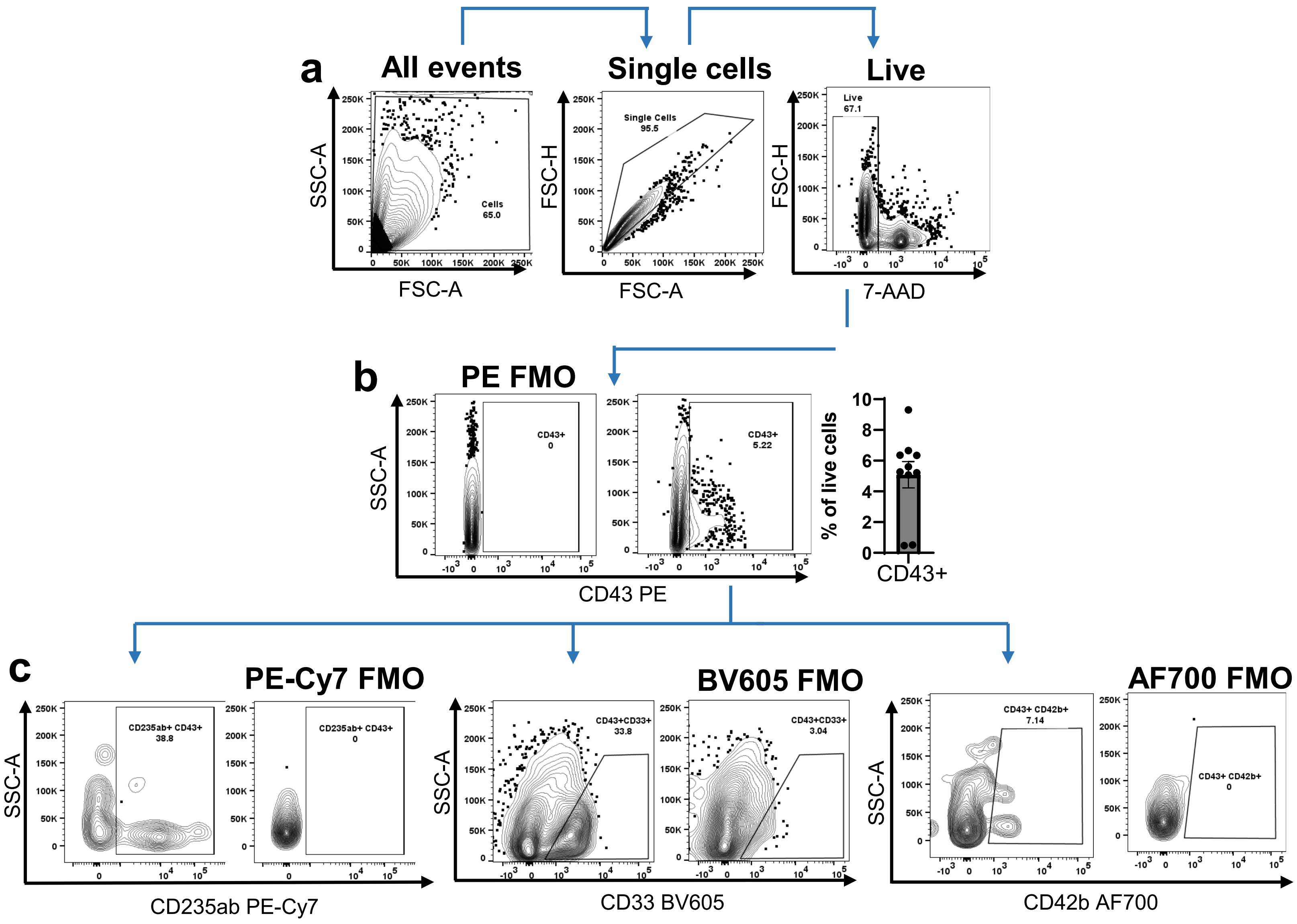

1) Supplementary Figure 1: Flow cytometry backgating strategies

A. Representative flow cytometry plots showing the gatings from all events to live cells. This gating strategy has been used for all flow cytometry data analysis.
B. Representative flow cytometry plots showing the gatings of $CD43^{+}$ compared to FMO control. This gating is used for flow cytometry plots in main figure 5J. Error bars are ± SEM. 10 biological replicates were used to make the bar plot.
C. Representative flow cytometry plots showing the $CD235ab^{+}$, $CD33^{+}$ and $CD42b^{+}$ cells pre-gated for $CD43^{+}$ with their respective FMO controls (attributed to the main figure 5J).

Flow cytometry backgatings and controls

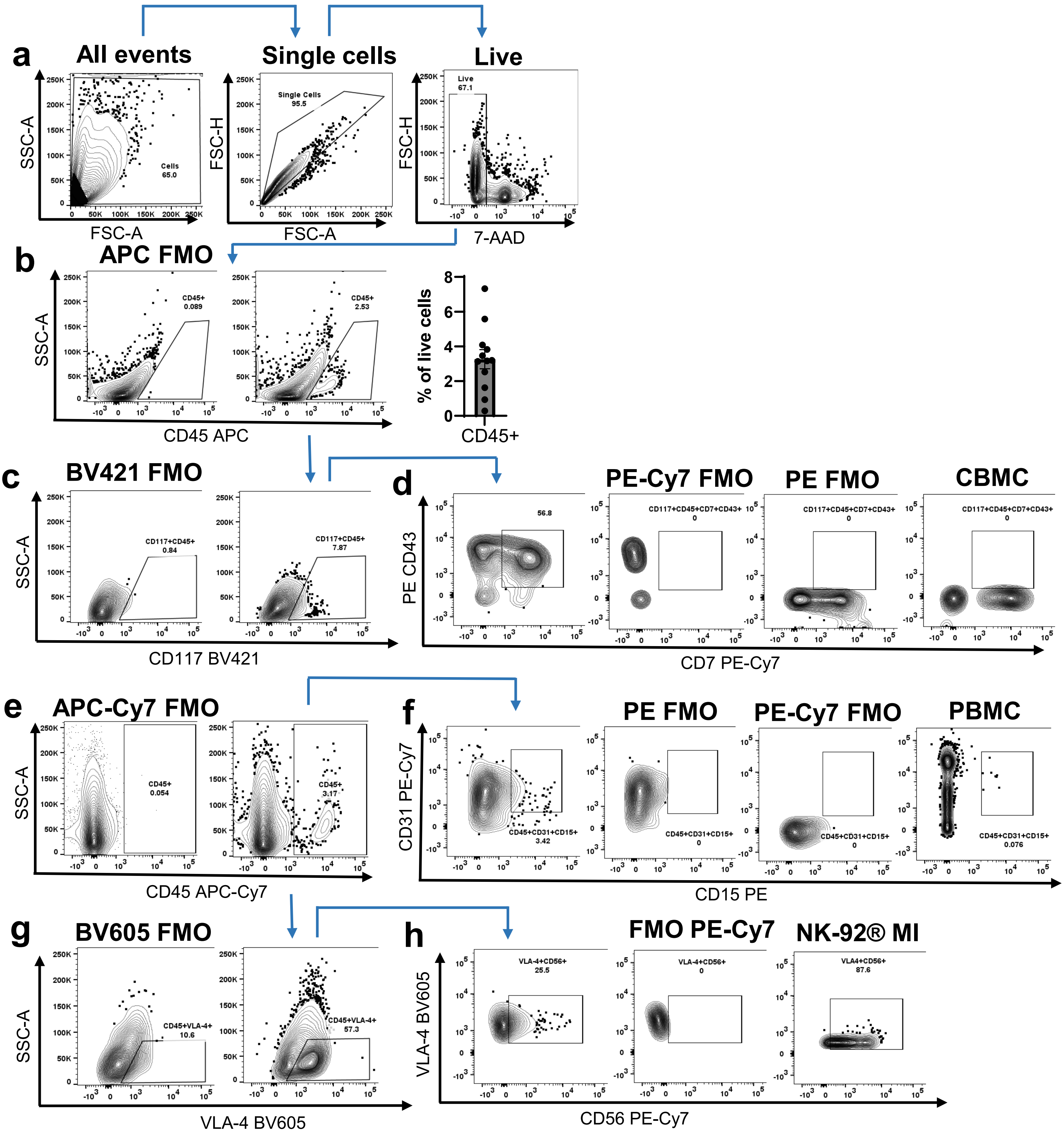

2) Supplementary Figure 2: Flow cytometry gating strategies

A. Representative flow cytometry plots showing the gatings from all events to live cells. This gating strategy has been used for all flow cytometry data analysis.
B. Representative flow cytometry plots showing the gating of $CD45^+$ compared to FMO control. This gating is used for initial gating after live cells gating for flow cytometry plot in main figure 5L. Error bars are ± SEM. 12 biological replicates was used for making the bar plot.
C. Representative flow cytometry plots showing the gating for $CD117^+$ cells pre-gated on $CD45^+$ cells in panel B. (attributed to the main figure 5L)
D. Representative flow cytometry plots showing the gating for $CD7^+$/$CD43^+$ cells pre-gated on $CD117^+$/ $CD45^+$ cells in panel C. CBMC: Cord blood mononuclear cells. (attributed to the main figure 5L)
E. Representative flow cytometry plots showing the gating of $CD45^+$ compared to FMO control. This gating is used for initial gating after live cells gating for flow cytometry plot in main figure 5K and 5M.
F. Representative flow cytometry plots showing the gating for $CD31^+$/$CD15^+$ cells pre-gated on $CD45^+$ cells in panel E. PBMC: Peripheral blood mononuclear cells. (attributed to the main figure 5K)
G. Representative flow cytometry plots showing the gating for VLA-4$^+$ cells pre-gated for $CD45^+$ cells shown in panel E. (attributed to the main figure 5M)
H. Representative flow cytometry plots showing the gating for CD56+ cells pre-gated for $CD45^+$ /VLA-4$^+$ cells shown in panel G. (attributed to the main figure 5M)

Primer sequences used for qRT-PCR

Gene Target	Sequence	Source
HBE	Forward: 5'-TCTGGCTACTCACTTTGGCAAGGA-3'<br>Reverse: 5'-TCACAGGAACACCTGCAAACTGGA-3'	Atkins et al. 2023 (reference 57)
HBG	Forward: 5'-TGGGAAATGTGCTGGTGACCGTTT-3'<br>Reverse: 5'-TCACAGGAACACCTGCAAACTGGA-3'	Atkins et al. 2023 (reference 57)
RNA18S5	Hs.PT.39a.22214856.g	IDT technologies

Supplementary Table 1: Oligonucleotides used for qRT-PCR
