# Supplementary material for: Modelling post-implantation human development to yolk sac blood emergence
Source: Nature. 2023 Dec 13;626(7998):367–76. doi: 10.1038/s41586-023-06914-8 (PMC10849971; doi:10.1038/s41586-023-06914-8)
Supplement: Supplementary file 2 — Reporting Summary [file 41586_2023_6914_MOESM2_ESM.pdf]

nature portfolio

Corresponding author(s): Mo Ebrahimkhani

Last updated by author(s): Nov 8, 2023

# Reporting Summary

Nature Portfolio wishes to improve the reproducibility of the work that we publish. This form provides structure for consistency and transparency in reporting. For further information on Nature Portfolio policies, see our Editorial Policies and the Editorial Policy Checklist.

## Statistics

For all statistical analyses, confirm that the following items are present in the figure legend, table legend, main text, or Methods section.

| n/a | Confirmed | |
|---|---|---|
| ☐ | ☒ | The exact sample size (*n*) for each experimental group/condition, given as a discrete number and unit of measurement |
| ☐ | ☒ | A statement on whether measurements were taken from distinct samples or whether the same sample was measured repeatedly |
| ☐ | ☒ | The statistical test(s) used AND whether they are one- or two-sided<br>*Only common tests should be described solely by name; describe more complex techniques in the Methods section.* |
| ☒ | ☐ | A description of all covariates tested |
| ☐ | ☒ | A description of any assumptions or corrections, such as tests of normality and adjustment for multiple comparisons |
| ☐ | ☒ | A full description of the statistical parameters including central tendency (e.g. means) or other basic estimates (e.g. regression coefficient) AND variation (e.g. standard deviation) or associated estimates of uncertainty (e.g. confidence intervals) |
| ☐ | ☒ | For null hypothesis testing, the test statistic (e.g. *F*, *t*, *r*) with confidence intervals, effect sizes, degrees of freedom and *P* value noted<br>*Give P values as exact values whenever suitable.* |
| ☒ | ☐ | For Bayesian analysis, information on the choice of priors and Markov chain Monte Carlo settings |
| ☒ | ☐ | For hierarchical and complex designs, identification of the appropriate level for tests and full reporting of outcomes |
| ☒ | ☐ | Estimates of effect sizes (e.g. Cohen's *d*, Pearson's *r*), indicating how they were calculated |

*Our web collection on statistics for biologists contains articles on many of the points above.*

## Software and code

Policy information about availability of computer code

| | |
|---|---|
| Data collection | Images were acquired using the EVOS M700 automated scanning microscope (software version 2.0.2094.0), Leica SP8 confocal microscope (Leica Application Suite X version 3.7.4), Incucyte S3 (software version v2019B), or Nikon A1 Confocal microscope (NIS Elements AR) and processed using ImageJ software (version 1.8.0_172). Any contrast adjustments were made in individual channels and applied evenly across the whole image in that channel. Contrast and color balance for color images was applied evenly across the whole image. Information for gene set enrichment analysis was collected using the Enrichr web server (https://maayanlab.cloud/Enrichr/, updated June 8, 2023) |
| Data analysis | Data was analyzed using: Graphpad Prism (v9), ImageJ (version 1.8.0_172), Imaris x64 (version 9.5.0), FlowJo (version 10.7.0), CellRanger (v2.1.0), R (3.6.3), Seurat (4.3.0), SeuratObject (4.1.3), sp (1.6-0), dplyr (1.0.10), devtools (2.3.2), mudata2 (1.1.2), plyr (1.8.8), ggplot2 (3.4.1), cowplot(1.1.1), patchwork (1.1.2), data.table (1.13.6), Matrix (1.5-3), caret (6.0-86), glmGamPoi (1.10.1), sctransform (0.3.5)<br>Wild-type cluster analysis was performed using an in-house Matlab pipeline generated in Matlab version 2020a. Hemoglobin analysis was performed using a pipeline developed in CellProfiler (version 4.2.5).<br>Scripts can be found at https://github.com/AmirAlavi/GATA6_R |

For manuscripts utilizing custom algorithms or software that are central to the research but not yet described in published literature, software must be made available to editors and reviewers. We strongly encourage code deposition in a community repository (e.g. GitHub). See the Nature Portfolio guidelines for submitting code & software for further information.

## Data

Policy information about availability of data

All manuscripts must include a data availability statement. This statement should provide the following information, where applicable:

- Accession codes, unique identifiers, or web links for publicly available datasets
- A description of any restrictions on data availability
- For clinical datasets or third party data, please ensure that the statement adheres to our policy

The sequencing reads, and single-cell expression matrices for all the single-cell RNA-seq data are submitted to NIH BioProject accession number PRJNA1035788. For hypergeometric analysis, we used publicly available data from Tyser et al. processed peri-implantation dataset E-MTAB-3929, Xiang et al. GSE136447 and Ma et al. GSE130114.
The datasets generated during and/or analyzed during the current study are available from the corresponding author on reasonable request. All scripts used have been deposited at https://github.com/AmirAlavi/GATA6_R

## Research involving human participants, their data, or biological material

Policy information about studies with human participants or human data. See also policy information about sex, gender (identity/presentation), and sexual orientation and race, ethnicity and racism.

| | |
|---|---|
| Reporting on sex and gender | No reportings or findings related to sex or gender were made or presented. |
| Reporting on race, ethnicity, or other socially relevant groupings | No reportings or findings related to race, ethnicity, or other groupings were made or presented. |
| Population characteristics | No human participants were used in this data. |
| Recruitment | No participants were recruited for this data. |
| Ethics oversight | The University of Pittsburgh Stem Cell Research Oversight board approved the study. |

Note that full information on the approval of the study protocol must also be provided in the manuscript.

# Field-specific reporting

Please select the one below that is the best fit for your research. If you are not sure, read the appropriate sections before making your selection.

☒ Life sciences ☐ Behavioural & social sciences ☐ Ecological, evolutionary & environmental sciences

For a reference copy of the document with all sections, see nature.com/documents/nr-reporting-summary-flat.pdf

# Life sciences study design

All studies must disclose on these points even when the disclosure is negative.

| | |
|---|---|
| Sample size | All experiments were conducted with multiple biological replicates, with the exact number described in the text. We did not predetermine sample sizes via statistical methods. The number of samples used in each experiment was selected to ensure data consistency/reproducibility and was based on the available resources. Sample size for single cell RNA-Seq was determined based on our estimation of the number of cells required to capture cell types of interest detected via immunofluorescence at the developmental stage. |
| Data exclusions | No samples were excluded. For computational analysis of samples, a crop of the central 9000 pixels, excluding the edges of the sample, was used. |
| Replication | The exact numbers of replicates for methods/analyses used are indicated in the respective figure legends. All replications were successful. Each individual experimental staining was repeated in at least two dishes and/or coverslips. Additionally, a subset of experiments were validated in two cell lines (PGP1 and PGP9), and by multiple investigators involved in this work. |
| Randomization | Embryoid samples developed in each culture well for each experiment were allocated to control and experimental groups in a random manner at seeding when different conditions (i.e. pathway inhibition) were present. |
| Blinding | For hypergeormetric analysis the person who did the analysis was not aware of the groups and identity of the samples and grouping. For small molecule treatment the identity of samples are sometimes obvious due to the morphological changes, hence blinding is not practical. Media changes cannot be performed with blinding as each group should receive the appropriate media. In all other experiments we had no relevant scientific reasons to conduct blinding as the experiments were mostly descriptive. |

# Reporting for specific materials, systems and methods

We require information from authors about some types of materials, experimental systems and methods used in many studies. Here, indicate whether each material, system or method listed is relevant to your study. If you are not sure if a list item applies to your research, read the appropriate section before selecting a response.

## Materials & experimental systems

| n/a | Involved in the study |
| --- | --- |
| ☐ | ☒ Antibodies |
| ☐ | ☒ Eukaryotic cell lines |
| ☒ | ☐ Palaeontology and archaeology |
| ☒ | ☐ Animals and other organisms |
| ☒ | ☐ Clinical data |
| ☒ | ☐ Dual use research of concern |
| ☒ | ☐ Plants |

## Methods

| n/a | Involved in the study |
| --- | --- |
| ☒ | ☐ ChIP-seq |
| ☐ | ☒ Flow cytometry |
| ☒ | ☐ MRI-based neuroimaging |

## Antibodies

Antibodies used

The following antibodies were used in this study. All primary antibodies for immunofluorescence were used at a dilution of 1:200. All secondary antibodies were used at a dilution of 1:400. All FACS antibodies were used at a dilution of 1:400. The specific clone for monoclonal antibodies is given in parentheses when applicable.

GFP Abcam (B-2) ab13970
GFP Aves Labs (Polyclonal) GFP-1020
NANOG R&D (Polyclonal) AF1997
PDGFRα Cell Signaling (D13C6) 5241S
OCT4 Cell Signaling (Polyclonal) mab2750
CD34 abcam (EP373Y) ab81289
CD43 R&D (290111) MAB2038
CD31 abcam (JC/70A) AB9498-1001
CD41 abcam (EPR4330) AB134131-1001
CD33 R&D (996810) MAB11371
CD42b R&D (Polyclonal) AF4067
CD235ab Biolegend (HIR2) 306602
TAL1 Santa Cruz (Polyclonal) sc12984
ERG abcam (EPR3864) ab92513
RUNX1 Santa Cruz (A-2) sc-365644
VEGFR2 R&D (89115) MAB3571
Hemoglobin R&D (Polyclonal) G-134-C
CX3CR1 abcam (Polyclonal) AB167571-1001
Hoechst 33342 Thermo Fisher H3570
Phalloidin-iFluor 405 AB176752-1001
PODXL Invitrogen (AB_2532205) 433140
ZO-1 Invitrogen (Polyclonal) 617300
LAMA1 Sigma-Aldritch (Polyclonal) SAB4501255
ISL1 Abcam (EP4182) ab109517
Phospho-SMAD1/5/8 Cell Signaling (D5B10) 13820T
Phospho-SMAD2 Cell Signaling (D27F4) 8828S
MIXL1 Invitrogen (Polyclonal) PA564903
TBXT R&D (Polyclonal) AF2085
CER1 abcam (Polyclonal) ab184133
VE-cad R&D (Polyclonal) AF938
Desmin R&D (Polyclonal) AF3844
FOXA2 Santa Cruz (H-8) sc-271104
PAX6 abcam (AD2.38) ab78545
NCAM abcam (EP2567Y) ab75813
HHEX R&D (2018B) MAB83771
GATA6 R&D (Polyclonal) AF1700
AP-2α Invitrogen (3B5) MA1-872

FACS Analysis:
CD34-APC Biolegend (581) 343510
CD31-PE/Cy7 Biolegend (WM59) 303118
CD42b-AF700 Biolegend (HIP1) 303928
CD33-BV605 Biolegend (P67.6) 366612
CD45-APC/Cy7 Biolegend (2D1) 368516
CD45-Pacific Blue Biolegend (2D1) 368539
CD45-APC Biolegend (HI30) 304012

CD43-PE Biolegend (CD43-10G7) 343203
CD235ab-PE/Cy7 (HIR2) Biolegend 306620
CD7-PE/Cy7 Biolegend (4H9/CD7) 395609
CD15-PE Biolegend (HI98) 301905
CD49d(VLA-4)-BV605 (9F10) Biolegend 304313
CD117-BV421 BD Biosciences (104D2) 563856
CD56-PE/Cy7 Biolegend (MEM-188) 304628

Secondary Antibodies for immunofluorescence:
Alexa Fluor® 594 AffiniPure Donkey Anti-Rabbit IgG (H+L) Jackson Immunoresearch 711-585-152
Alexa Fluor® 647 AffiniPure Donkey Anti-Goat IgG (H+L) Jackson Immunoresearch 705-605-147
Alexa Fluor® 647 AffiniPure Donkey Anti-Mouse IgG (H+L) Jackson Immunoresearch 715-605-151
Alexa Fluor® 647 AffiniPure Donkey Anti-Sheep IgG (H+L) Jackson Immunoresearch 713-605-147
Alexa Fluor® 488 AffiniPure Donkey Anti-Chicken IgY (IgG) (H+L) Jackson Immunoresearch 703-545-155

Validation

All antibodies were obtained from commercial sources. Detailed validation statements are available on the corresponding manufacturers' websites, as provided below:

GFP Abcam (B-2) ab13970, referenced in 3182 publications: https://www.abcam.com/products/primary-antibodies/gfp-antibody-ab13970.html

GFP Aves Labs (Polyclonal) GFP-1020, referenced in 1782 publications: https://www.aveslabs.com/products/anti-green-fluorescent-protein-antibody-gfp

NANOG R&D (Polyclonal) AF1997, referenced in 207 publications: https://www.rndsystems.com/products/human-nanog-antibody_af1997

PDGFRα Cell Signaling (D13C6) 5241S, referenced in 60 publications: https://www.cellsignal.com/products/primary-antibodies/pdgf-receptor-a-d13c6-xp-rabbit-mab/5241

OCT4 Cell Signaling (Polyclonal) mab2750, cited in 312 publications: https://www.cellsignal.com/products/primary-antibodies/oct-4-antibody/2750

CD34 abcam (EP373Y) ab81289, referenced in 499 publications: https://www.abcam.com/products/primary-antibodies/cd34-antibody-ep373y-ab81289.html

CD43 R&D (290111) MAB2038, referenced in 1 publication: https://www.rndsystems.com/products/human-cd43-antibody-290111_mab2038

CD31 abcam (JC/70A) AB9498-1001, referenced in 210 publications: https://www.abcam.com/products/primary-antibodies/cd31-antibody-jc70a-ab9498.html

CD41 abcam (EPR4330) AB134131-1001, referenced in 20 publications: https://www.abcam.com/products/primary-antibodies/cd41-antibody-epr4330-ab134131.html

CD33 R&D (996810) MAB11371, https://www.rndsystems.com/products/human-siglec-3-cd33-antibody-996810_mab11371

CD42b R&D (Polyclonal) AF4067, referenced in 2 publications: https://www.rndsystems.com/products/human-cd42b-gpib-alpha-antibody_af4067

CD235ab Biolegend (HIR2) 306602, referenced in 7 publications: https://www.biolegend.com/en-us/products/purified-anti-human-cd235ab-antibody-743

TAL1 Santa Cruz (Polyclonal) sc12984, referenced in 12 publications: https://www.scbt.com/p/tal1-antibody-c-21

ERG abcam (EPR3864) ab92513, referenced in 201 publications: https://www.abcam.com/products/primary-antibodies/erg-antibody-epr3864-ab92513.html

RUNX1 Santa Cruz (A-2) sc-365644, referenced in 47 publications: https://www.scbt.com/p/runx1-antibody-a-2

VEGFR2 R&D (89115) MAB3571, referenced in 17 publications: https://www.rndsystems.com/products/human-vegfr2-kdr-flk-1-antibody-89115_mab3571

Hemoglobin R&D (Polyclonal) G-134-C, referenced in 2 publications: https://www.rndsystems.com/products/human-hemoglobin-antibody_g-134-c

CX3CR1 abcam (Polyclonal) AB167571-1001: https://www.abcam.com/products/primary-antibodies/cx3cr1-antibody-ab167571.html

Hoechst 33342 Thermo Fisher H3570: https://www.thermofisher.com/order/catalog/product/H3570?SID=srch-srp-H3570

Phalloidin-iFluor 405 AB176752-1001, referenced in 14 publications: https://www.abcam.com/products/chip-kits/phalloidin-ifluor-405-reagent-ab176752.html

PODXL Invitrogen (AB_2532205) 433140: https://www.thermofisher.com/antibody/product/PODXL-Antibody-Monoclonal/433140

ZO-1 Invitrogen (Polyclonal) 617300, referenced in 765 publications: https://www.thermofisher.com/antibody/product/ZO-1-

Antibody-Polyclonal/61-7300

LAMA1 Sigma-Aldritch (Polyclonal) SAB4501255: https://www.sigmaaldrich.com/US/en/product/sigma/sab4501255

ISL1 Abcam (EP4182) ab109517, referenced in 52 publications: https://www.abcam.com/products/primary-antibodies/islet-1-antibody-ep4182-neural-stem-cell-marker-ab109517.html

Phospho-SMAD1/5/8 Cell Signaling (D5B10) 13820T, referenced in 412 publications: https://www.cellsignal.com/products/primary-antibodies/phospho-smad1-ser463-465-smad5-ser463-465-smad9-ser465-467-d5b10-rabbit-mab/13820

Phospho-SMAD2 Cell Signaling (D27F4) 8828S, referenced in 676 publications: https://www.cellsignal.com/products/primary-antibodies/phospho-smad2-ser465-467-smad3-ser423-425-d27f4-rabbit-mab/8828

MIXL1 Invitrogen (Polyclonal) PA564903, referenced in 2 publications: https://www.thermofisher.com/antibody/product/MIXL1-Antibody-Polyclonal/PA5-64903

TBXT R&D (Polyclonal) AF2085, referenced in 145 publications: https://www.rndsystems.com/products/human-mouse-brachyury-antibody_af2085

CER1 abcam (Polyclonal) ab184133: https://www.abcam.com/products/primary-antibodies/cer1-antibody-ab184133.html

VE-cad R&D (Polyclonal) AF938, referenced in 17 publications: https://www.rndsystems.com/products/human-ve-cadherin-antibody_af938

Desmin R&D (Polyclonal) AF3844, referenced in 16 publications: https://www.rndsystems.com/products/human-mouse-desmin-antibody_af3844

FOXA2 Santa Cruz (H-8) sc-271104, referenced in 1 publication: https://www.scbt.com/p/hnf-3beta-antibody-h-8?requestFrom=search

PAX6 abcam (AD2.38) ab78545, referenced in 44 publications: https://www.abcam.com/products/primary-antibodies/pax6-antibody-ad238-ab78545.html

NCAM abcam (EP2567Y) ab75813 , referenced in 35 publications: https://www.abcam.com/products/primary-antibodies/ncam1-antibody-ep2567y-ab75813.html

HHEX R&D (2018B) MAB83771, referenced in 6 publications: https://www.rndsystems.com/products/human-mouse-rat-hhex-antibody-2018b_mab83771

GATA6 R&D (Polyclonal) AF1700, referenced in 61 publications: https://www.rndsystems.com/products/human-gata-6-antibody_af1700

AP-2α Invitrogen (3B5) MA1-872, referenced in 3 publications: https://www.thermofisher.com/antibody/product/AP2-alpha-Antibody-clone-3B5-Monoclonal/MA1-872

CD34-APC Biolegend (581) 343510, referenced in 18 publications: https://www.biolegend.com/en-us/products/apc-anti-human-cd34-antibody-6090

CD31-PE/Cy7 Biolegend (WM59) 303118, referenced in 18 publications: https://www.biolegend.com/en-us/products/pe-cyanine7-anti-human-cd31-antibody-6124

CD42b-AF700 Biolegend (HIP1) 303928: https://www.biolegend.com/en-us/products/alexa-fluor-700-anti-human-cd42b-antibody-14765

CD33-BV605 Biolegend (P67.6) 366612, referenced in 1 publication: https://www.biolegend.com/en-us/products/brilliant-violet-605-anti-human-cd33-antibody-12255

CD45-APC/Cy7 Biolegend (2D1) 368516, referenced in 30 publications: https://www.biolegend.com/en-us/products/apc-cyanine7-anti-human-cd45-antibody-12400

CD45-Pacific Blue Biolegend (2D1) 368539, referenced in 11 publications: https://www.biolegend.com/en-us/products/pacific-blue-anti-human-cd45-antibody-14908

CD45-APC Biolegend (HI30) 304012, referenced in 74 publications: https://www.biolegend.com/en-us/products/apc-anti-human-cd45-antibody-705

CD43-PE Biolegend (CD43-10G7) 343203, referenced in 4 publications: https://www.biolegend.com/en-us/products/pe-anti-human-cd43-antibody-6011

CD235ab-PE/Cy7 (HIR2) Biolegend 306620, referenced in 7 publications: https://www.biolegend.com/en-us/products/purified-anti-human-cd235ab-antibody-743

CD7-PE/Cy7 Biolegend (4H9/CD7) 395609: https://www.biolegend.com/en-us/products/pe-cyanine7-anti-human-cd7-antibody-21684

CD15-PE Biolegend (HI98) 301905, referenced in 27 publications: https://www.biolegend.com/en-us/products/pe-anti-human-cd15-

ssea-1-antibody-713

CD49d(VLA-4)-BV605 (9F10) Biolegend 304313, referenced in 16 publications: https://www.biolegend.com/en-us/products/pe-cyanine7-anti-human-cd49d-antibody-6776

CD117-BV421 BD Biosciences (104D2) 563856: https://www.bdbiosciences.com/en-us/products/reagents/flow-cytometry-reagents/research-reagents/single-color-antibodies-ruo/bv421-mouse-anti-human-cd117.563856

CD56-PE/Cy7 Biolegend (MEM-188) 304628, referenced in 7 publications: https://www.biolegend.com/en-us/products/pe-cyanine7-anti-human-cd56-ncam-antibody-4584

# Eukaryotic cell lines

Policy information about cell lines and Sex and Gender in Research

| | |
|---|---|
| Cell line source(s) | PGP1 and PGP9 parental cell lines were supplied through an MTA from the Weiss lab at MIT, and can be obtained from Coriell. |
| Authentication | PGP1 and PGP9 hiPSC lines were authenticated in-house via immunostaining for pluripotency markers (OCT4, NANOG, SOX2) and successful differentiation to the three germ layers.<br>Engineered cell lines were assessed for functionality via detection of expression of GATA6 and EGFP. |
| Mycoplasma contamination | All cell lines tested negative for mycoplasma contamination. |
| Commonly misidentified lines<br>(See ICLAC register) | No commonly misidentified cell lines listed by ICLAC were used in this work. |

# Flow Cytometry

## Plots

Confirm that:

☒ The axis labels state the marker and fluorochrome used (e.g. CD4-FITC).

☒ The axis scales are clearly visible. Include numbers along axes only for bottom left plot of group (a 'group' is an analysis of identical markers).

☒ All plots are contour plots with outliers or pseudocolor plots.

☒ A numerical value for number of cells or percentage (with statistics) is provided.

## Methodology

| | |
|---|---|
| Sample preparation | Cultures were treated for 45 minutes with Collagenase C solution (3 mg/ml Stem Cell Technologies) followed by 15 minutes treatment with Accuatse (Sigma) and filtered through 40μm filters (Thermo Fisher) to make the single cell suspension. FC block solution (Thermo Fischer) was added to the samples followed by 10 minutes incubation on ice. Next, the antibody mix (1:400) was added to the samples followed by 30 minutes incubation on ice. Cells were analyzed using an LSR II flow cytometer (BD Bioscience) using 7-AAD (BD Pharmingen) for dead cell staining. |
| Instrument | Cells were analyzed using an LSR II or Fortessa flow cytometer (BD Biosciences) |
| Software | FACSDiva (BD Biosciences) software suite was used for collection. FlowJo software (version 10.7.0) was used for flow cytometry analysis. |
| Cell population abundance | Abundance of distinct cell populations of interest was determined using appropriate negative controls. |
| Gating strategy | Standard gating settings were used. Cell debris was excluded via an SSC-A vs FSC-A gate; aggregates were excluded by comparing FSC-A and FSC-H; dead cells were gated out using the 7-AAD stain to identify positive cells. The final gating is available in Supplementary Information 1. |

☒ Tick this box to confirm that a figure exemplifying the gating strategy is provided in the Supplementary Information.
